# Decoding the full picture of Raf1 function based on its interacting proteins

**DOI:** 10.18632/oncotarget.19353

**Published:** 2017-07-18

**Authors:** Ying Huang, Xiao-Xi Guo, Bing Han, Xu-Min Zhang, Su An, Xin-Yu Zhang, Yang Yang, Ying Liu, Qian Hao, Tian-Rui Xu

**Affiliations:** ^1^ Faculty of Life Science and Technology, Kunming University of Science and Technology, Kunming, China; ^2^ Faculty of Environmental Science and Engineering, Kunming University of Science and Technology, Kunming, China; ^3^ Institute of Biomedical Sciences, Minhang Hospital, Fudan University, Shanghai, China; ^4^ State Key Laboratory of Genetic Engineering, Department of Biochemistry, School of Life Sciences, Fudan University, Shanghai, China

**Keywords:** Raf1, interacting protein, cancer, cell signaling, oncological target

## Abstract

Raf1 is a member of the Raf kinase family and regulates many fundamental cell processes, including proliferation, differentiation, apoptosis, motility, and metabolism. However, the functions of Raf1 have not been completely elucidated. To better understand Raf1 function, we investigated the proteins that interacted with Raf1. We identified 198 Raf1 interacting proteins and our data suggested that Raf1 may regulate cell processes through these interactions. These interaction partners were involved in all ten hallmarks of cancer, suggesting that Raf1 is involved in different aspects of carcinogenesis. In addition, we showed that Raf1 interacting proteins were enriched in six signaling pathways and many human diseases. The interaction partners identified in this study may represent oncological candidates for future investigations into Raf1 function. Our findings have provided an overview of Raf1 function from a systems biology perspective.

## INTRODUCTION

As an activator of mitogen-activated protein kinase (MAPK)/ERK kinase (MEK) pathway and an effector of Ras, Raf is involved in many fundamental cellular processes such as cell proliferation, differentiation, cell death and survival, metabolism and motility [1–3]. The mammalian Raf kinase family includes A-Raf, B-Raf, and Raf1 (C-Raf). These proteins all have auto-inhibitory, regulatory, and catalytic domains, and contain multiple phosphorylation sites [4]. Among the three members, Raf1 has drawn the most attention since it was identified 30 years ago [5]. Raf1 has been widely reported as a key effector of the small G protein Ras, and after activation, Raf can phosphorylate MEK, which then activates ERK. ERK then phosphorylates an impressive roster of membrane, cytosolic, and nuclear targets to regulate numerous cell functions [6].

However, unlike B-Raf, Raf1 is not essential for ERK activation. During tumorigenesis, Raf1 interacts with different proteins to allow cross-talk between signaling pathways [7]. Protein phosphatase 2A (PP2A)/PP1 and members of the 14-3-3 family can interact with Raf1 to control its enzymatic activity [8, 9]. Heat shock protein 90 (HSP90) and its co-chaperone CDC37 have also been identified as Raf-associated proteins that are crucial for the maturation and activation of Raf1 [10]. Further research has identified more proteins that interact with and regulate the activity of Raf1. These proteins include p21-activated kinase (PAK3), serine/threonine kinase 3 (STK3), and protein kinase C (PKC) [11, 12]. However, the proteins interacting with Raf1 have not been fully elucidated.

To explore the biological functions of Raf1, we aimed to create an overview of the Raf1 interactome. We enriched Raf1-interacting proteins under stable, MAPK-inactive conditions using co-immunoprecipitation. Then, we identified these interacting proteins using liquid chromatography–mass spectrometry (LC-MS). We uncovered 198 Raf1-interacting proteins and confirmed 12 of these interactions by western blotting. Gene ontology (GO) and pathway enrichment analysis indicated that these Raf1-protein interactions regulated six signaling pathways, and were involved in the ten known hallmarks of cancer. Our findings have deepened our understanding of Raf1 function, and offer potential oncological targets for studying specific Raf1 functions in the future.

## RESULTS

### Raf1 co-immunoprecipitated with 198 proteins

**Figure 1:**
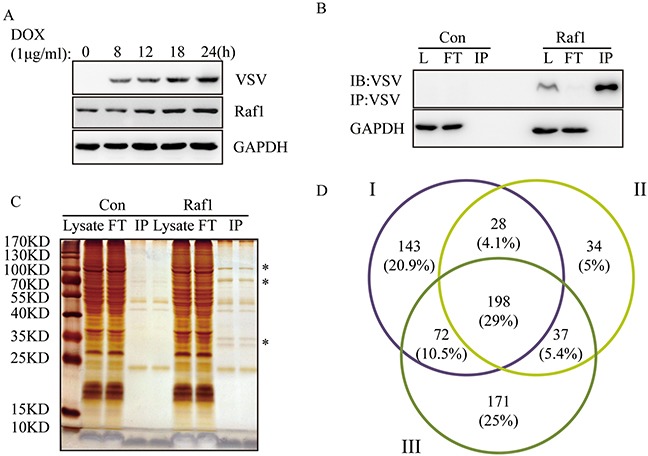
Identification of Raf1 interacting proteins in three independent experiments **(A)** Expression of VSV-tagged-Raf1 in Flp-In To-REx HEK293 cells. Cells were induced with 1μg/ml of doxycycline for 8, 12, 18, and 24 hours. VSV-RAF-1 and Raf1 (exogenous and endogenous) were detected using anti-VSV and anti-Raf1 antibodies, respectively. **(B)** VSV-Raf1 was immunoprecipitated with mouse anti-VSV-G antibody covalently bound to agarose beads and detected using an VSV antibody (L: lysate, FT: flow through). Flp-In To-REx cells not expressing VSV-Raf1 were used as negative controls (Con). **(C)** Samples from (B) were resolved by SDS-PAGE and observed by silver staining. Flp-In To-REx cells not expressing VSV-Raf1 were used as negative controls (Con). Specific Raf1 interacting proteins are indicated by a star*, two bands of IP were the some samples with two loading repeats (10μl/5μl). **(D)** Venn diagram corresponding to proteins identified in three independent Raf1 immunoprecipitations. The overlapping 198 proteins correspond to proteins common to the three data sets (http://bioinfogp.cnb.csic.es/tools/venny/).

To identify Raf1 interacting proteins, we induced VSV-Raf1 expression with doxycycline in Flp-in To-REx HEK293 cells containing VSV-Raf1 (Figure 1A). After doxycycline induction, cell lysates were precipitated with anti-VSV-glycoprotein-agarose beads. Exogenous VSV-Raf1 was effectively pulled down and very little VSV-Raf1 remained in the flow through (FT) line (Figure 1B). Immunoprecipitated proteins were separated by SDS-PAGE then observed by silver staining. As shown in Figure 1C, most of the Raf1 specific interacting proteins are concentrated in the ranges of 25∼35 KDa and 70∼130 KDa. Immunoprecipitated proteins were analyzed by LC−MS. In three independent experiments, 441, 297, and 478 Raf1 interacting proteins were identified. Comparison of these three data sets (Figure 1D) revealed 198 common putative Raf1 interacting proteins. This number was comparable with previously published work [13, 14]. The full list and details of these 198 proteins is presented in Supplementary Table 1.

### 182 out of these 198 proteins are novel Raf1-interacting proteins

**Figure 2:**
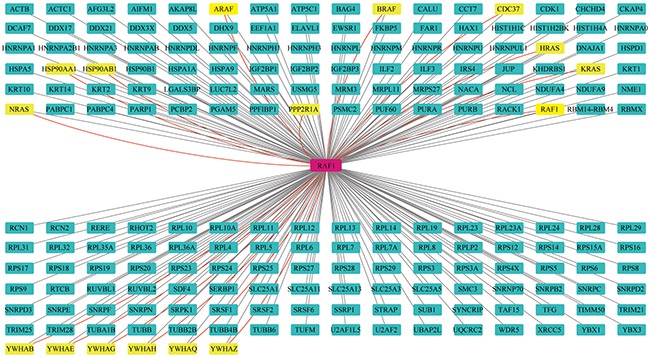
Interaction network of proteins from the Raf1 interactome Novel interaction partners are identified by black lines and green nodes. Previously identified interaction partners are shown as red lines and yellow nodes.

Based on the Raf1 mapping information obtained from the STRING database, we exported 69 experimentally validated interacting proteins that had a combined score of >0.9 (Supplementary Table 2). These interacting proteins included H-RAS, N-RAS, K-RAS, AKT, and PAK1/2/3/4. We found that 16 of our 198 identified interaction partners had been reported previously. These were *YWHAB, YWHAE, YWHAG, YWHAH, YWHAQ, YWHAZ, HSP90AA1, HSP90AB1, CDC37, KRAS, NRAS, HRAS, PPP2R1A*, and *BRAF* (gene names) (Figure 2).

### Western blotting confirmed the reliability of Raf1-interacting proteins

**Figure 3:**
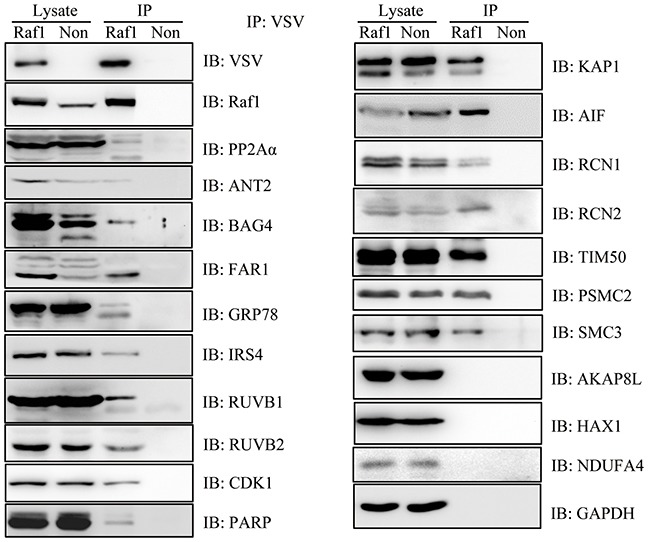
Western blotting confirmed the interaction of 12 proteins with Raf1 Twenty-four hours after doxycycline induction, VSV-Raf1 protein was immunoprecipitated from Flp-In To-Rex HEK293 cells expressing VSV-Raf1 (Raf1) with anti-VSV-G agarose beads. VSV-Raf1 associated proteins were detected by western blotting using the indicated antibodies. Flp-In To-Rex HEK293 cells containing the pcDNA5 empty vector (Non) were used as controls. GAPDH was immunoprecipitated as a negative binding control (LC-MS data showed that GAPDH did not interact with Raf1) and as a reference of equal loading.

To confirm the reliability of these 198 proteins, we selected 20 interacting proteins of different functions and examined the interaction by western blotting. We were able to confirm an interaction with Raf1 for 17 out of 20 (∼85%) selected Raf1 interacting proteins. We could not detect an interaction of AKAP8L, NDUFA4 or HAX1 with Raf1 by western blotting (Figure 3).

### Novel Raf1 interacting proteins were involved in many biological processes and distributed in different cellular components

DAVID software was utilized to identify GO categories and KEGG pathways in the 198 Raf1-interacting proteins. GO analysis showed that certain MFs were enriched for Raf1 interacting proteins, including pyrophosphatase activity, enzyme binding, protein kinase binding, GTPase activity, and heat shock protein binding. In addition, Raf1 interacting proteins were enriched in BPs, including positive regulation of metabolism, programmed cell death, cellular stress responses, chromosome organization, and cell-cell adhesion. In addition, GO CC analysis demonstrated significant enrichment for cell junction, mitochondrion, methyltransferase complex, nucleolus, and extracellular matrix proteins (Supplementary Tables 3–5). In Ingenuity Diseases and Bio Functions analysis, Raf1 interacting proteins were enriched to 15 bio functions which were all covered by GO analysis results. However, inductions and descriptions are slightly different between these two analyses (Supplementary Table 7).

### Raf1 interacting proteins contribute to all hallmarks of cancer

Ten hallmarks of cancer have been described by Hanahan and Weinberg (outlined in Table 1) [15, 16]. These describe the acquired biological capabilities of cancer cells. We investigated the contribution of Raf1 interacting proteins to the hallmarks and states of cancer based on GO enrichment (BPs and MFs). Table 1 shows specific GO terms that apply to cancer hallmarks, including regeneration (GO: 0031099) and the ERBB2 signaling pathway (GO:0038128), which correlate with “evading growth suppressors”. We confirmed that Raf1 interacting proteins are involved in all ten hallmarks of cancer.

**Table 1:** Classification of Raf1 interacting proteins based on the hallmarks of cancer

Hallmark	Term	GO term and function
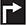	Sustaining proliferative signaling	GO: 0030518∼intracellular steroid hormone receptor signaling pathwayGO: 0038128∼ERBB2 signaling pathwayGO: 0048545∼response to steroid hormone
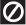	Evading growth suppression	GO: 0031099∼regenerationGO: 0038128∼ERBB2 signaling pathway
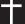	Resisting cell death	GO: 0012501∼programmed cell deathGO: 0010506∼regulation of autophagy
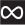	Enabling replicative immortality	GO: 1903047∼mitotic cell cycle processGO: 0000723∼telomere maintenanceGO: 0010833∼telomere maintenance via telomere lengthening
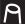	Inducing angiogenesis	GO: 0031100∼organ regeneration
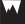	Activating invasion and metastasis	GO: 0098609∼cell-cell adhesionGO: 0045296∼cadherin binding
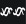	Genome instability and mutation	GO: 0006281∼DNA repairGO: 0051276∼chromosome organization
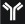	Avoiding immune destruction	GO: 0002253∼activation of immune response
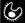	Tumor-promoting inflammation	GO: 0071353∼cellular response to interleukin-4GO: 0001817∼regulation of cytokine productionGO: 1902187∼negative regulation of viral release from host cell
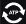	Deregulating cellular energetics	GO: 0044248∼cellular catabolic processGO: 0046034∼ATP metabolic process

### Raf1 interacting proteins were enriched in 6 signal pathways and 15 diseases

Supplementary Table 6 shows the pathways in which Raf1 interacting proteins were most significantly enriched based on KEGG pathway analysis. The results were divided into three categories: biological functions, cellular signaling pathways, and diseases. Interacting proteins were most enriched in the following biological functions: cell cycle, oocyte meiosis, gap junctions, long-term potentiation, long-term depression, progesterone-mediated oocyte maturation, and pathogenic *Escherichia coli* infection. Raf1 interacting proteins were significantly enriched in the ErbB, FoxO, PI3K-Akt, Hippo, insulin, and estrogen signaling pathways. Regarding human diseases, Raf1-interacting proteins were mainly enriched in different cancers, including endometrial cancer, renal cell carcinoma, glioma, prostate cancer, thyroid cancer, melanoma, bladder cancer, leukemia, and non-small cell lung cancer. Viral infections were also enriched, including hepatitis C/B, Epstein-Barr virus, and viral carcinogenesis.

According to Ingenuity Canonical Pathways analysis and Ingenuity Diseases analysis, Raf1 interacting proteins were enriched to 157 signal pathways and 18 diseases due to its broad classification criteria (Supplementary Tables 7, 9).

## DISCUSSION

The goal of the present work was to decode Raf1 functions and get a global review of its biological role in cell processes, especially in carcinogenesis. We identified 198 Raf1 interacting proteins using *in vitro* VSV-tagging (Figure 2) and 14 of these had been reported previously reported to interact with Raf1. Among these 14 proteins, six belong to the 14-3-3 family (gene names: *YWHAB*, *YWHAE*, *YWHAG*, *YWHAH*, *YWHAQ*, and *YWHAZ*), which is well known to regulate Raf1 activity by phosphorylation and which is a highly conserved protein family that regulates many cellular processes such as proliferation, differentiation, apoptosis, and the cell cycle [17]. Three of the 14 previously identified binding proteins belong to the molecular chaperones (gene name: *HSP90AA1*, *HSP90AB1*, and *CDC37*), which are necessary for stabilizing the tertiary structure of Raf1 [18]. As the signal generator of Raf1, K/N/HRAS proteins were found to interact with Raf1 at high abundance in the interaction databases. The protein phosphatase PP2Aα, which is responsible for Raf1 inactivation, was also identified as an important Raf1-interacting protein [19]. The activation of Raf1 relies on Ras-dependent dimerization, including both homo- and hetero-dimerization [20]. Thus, we have also detected all three Raf variants among the Raf1-interacting proteins. Actually, two addition Raf1-interacting proteins recorded in Ingenuity Knowledge Base are A-Raf and Raf1 itself (Supplementary Table 8). These data suggest we may find new Raf1 functions from newly identified 182 proteins.

We verified our LC-MS data by confirming the interaction of selected binding partners with Raf1 by western blotting. To avoid selection bias, we chose proteins with different cellular locations, including the plasma membrane (IRS4, BAG4, CDK1 and PSMC2), mitochondria (AIF and ANT2), endoplasmic reticulum (GRP78 and RCN1/2), nucleus (KAP1, RUVB1/2, SMC3, PARP and TIM50), and peroxisomes (FAR1). In addition, selected proteins had independent roles in apoptosis, cell metabolism, cell proliferation, cell adhesion, DNA repair, protein scaffolding, and embryonic development [21–29]. Furthermore, we had access to good quality antibodies to detect these proteins. We confirmed an interaction with Raf1 for all selected proteins except AKAP8L, NDUFA4 and HAX1. This may be explained by the low sensitivity of the western blotting assay.

GO enrichment analysis revealed that many Raf1 interacting partners are involved in programmed cell death, cellular stress responses, and cell-cell adhesion. These findings may help to elucidate how Raf1 regulates these processes. We also found that Raf1 interacts with FAR1 and IRS4, suggesting that Raf1 may participate in lipid metabolism by protein interacting [28]. KEGG pathway analysis (Supplementary Table 6) indicated that Raf1 is involved in 10 biological functions, six cell signaling pathways, and 14 human diseases. Some of these findings were not reported previously and may provide novel insights into Raf1 functions.

To gain further insight into the role of Raf1 in cancer, we investigated whether the identified Raf1 interacting proteins are involved in the ten hallmarks of cancer (described in Table 1). Our findings showed that Raf1 sustains cell proliferation through interactions with proteins involved in hormone responses, growth signal transduction, and evasion of growth suppressors. Furthermore, Raf1 promotes resistance to apoptosis and autophagy in cancer cells, and enhances replicative immortality by regulating telomere maintenance. Raf1 has also been implicated in organ regeneration, therefore may induce angiogenesis, which is essential for tumor growth. The invasion and metastasis of cancer cells is controlled by changes in cell-cell adhesion. Therefore, the regulation of cell-cell adhesion by Raf1-interacting proteins may explain how Raf1 promotes tumor malignancy. Genome instability and gene mutations are hallmarks of cancer. Our findings indicated that Raf1 can promote DNA repair and chromosome organization through interactions with novel binding partners. We also observed that Raf1 interacting partners are involved in immune response activation and the response to interleukins. These interactions may allow host cells to avoid immune destruction and respond to tumor-promoting inflammation. Furthermore, Raf1 interactions control cellular catabolism and ATP metabolism to maintain the strong proliferative ability of cancer cells. Taken together, these findings suggest that Raf1 plays a more widespread role in carcinogenesis than was previously reported.

KEGG pathway analysis showed that novel Raf1-interacting proteins are also involved in cell signaling pathways and the development of various diseases, including endometrial cancer, which has not been reported previously. We found that the Raf1-interacting proteins *AIFM1, DDX17, DDX5, DNAJA1, HNRNPU, HSPD1, KRAS, PARP1, RBM14, RPL32*, and *YWHAH* (gene name) may contribute to the response to steroid hormones, especially estrogen. This suggests a mechanism for Raf1 in endometrial cancer.

In conclusion, we have identified 198 Raf1 interacting proteins and verified 17 of these by western blotting. The identified Raf1 interacting proteins were involved in all hallmarks of cancer, highlighting the importance of Raf1 in carcinogenesis. Much remains to be learned about Raf1 functions and the present study has identified putative candidates for future investigations. Furthermore, our findings provide a useful overview of Raf1 function from a systematic biology perspective.

## MATERIALS AND METHODS

### Materials

Doxycycline, monoclonal mouse anti-VSV-G antibody, and anti-VSV-G agarose beads were purchased from Sigma-Aldrich (Gillingham, United Kingdom). Lipofectamine 2000 transfection reagent was purchased from Invitrogen (Paisley, United Kingdom). Protease inhibitor cocktail tablets were purchased from Millipore (Massachusetts, United States). Anti-BAG4, anti-FAR1, anti-PP2Aα, anti-TIM50, anti-AKAP8L, anti-PSMC2, anti-SMC3 and anti-GAPDH antibodies were purchased from Santa Cruz (Heidelberg, Germany). Anti-Raf1, anti-AIF, anti-HAX1, and anti-GRP78 antibodies were purchased from BD Biosciences (Oxford, United Kingdom). The anti-ANT2 antibody was purchased from Cell Signaling Technology (Hitchin, Hertfordshire, United Kingdom). Anti-IRS4, anti-KAP1, anti-RCN1, anti-NDUFA4 and anti-RCN2 antibodies were purchased from Abcam (Cambridge, United Kingdom). Anti-VSV antibody was from sigma Sigma-Aldrich (Gillingham, United Kingdom). Anti-RUVB1 and anti- RUVB 2 antibodies were from Proteintech (Illinois, United States).

### Cell culture

Flp-In To-Rex HEK293 cells were maintained in Dulbecco’s modified Eagle medium (DMEM) supplemented with 10% (vol/vol) fetal bovine serum (FBS), 100 μg/mL zeocin, and 15 μg/mL blasticidin. Stable Flp-In To-Rex HEK293 cells inducibly expressing VSV-Raf1 were maintained in DMEM supplemented with 10% (vol/vol) FBS, 50 μg/mL hygromycin, and 15 μg/mL blasticidin.

### Generation of stable Flp-In TO-REx HEK293 cells inducibly expressing VSV-Raf1

VSV-Raf1 was amplified by PCR and inserted between *Bam*HI and *Xho*I sites of pcDNA5/FRT/TO. The primers were as follows: Forward (VSV-G sequence and *Bam*HI site underlined) CG GGATCC GCC ACC ATG TAC ACC GAT ATA GAG ATG AAC CGC CTT GGA AAG GAG CACAT ACA GGG AGC TTG GAA; reverse (*Xho*I site underlined) CCG CTCGAG CTA GAA GAC AGG CAG CCT CGG. To generate stable Flp-In To-Rex HEK293 cells that inducibly express VSV-Raf1, cells were transfected with a mixture of VSV-Raf1 cDNA in pcDNA5/FRT/TO vector and the pOG44 vector (1:9) using Lipofectamine 2000 transfection reagent, according to the manufacturer’s instructions. Resistant clones were selected by replacing zeocin with 200 μg/mL hygromycin B. VSV-Raf1 expression was induced by treating cells with 1 μg/mL doxycycline for 24 h and confirmed by western blotting using an anti-VSV antibody.

### Identification of VSV-Raf1-interacting proteins by mass spectrometry

VSV-Raf1 was induced by 1 μg of doxycycline/ml in Flp-in To-REx HEK293 cells. After 24 hours of induction, cells were harvested and resuspended in immunoprecipitation buffer (150 mM NaCl, 0.01 mM NaPO_4_, 2 mM EDTA, 0.5% Triton X-100, 5% glycerol, and protease inhibitor cocktail tablets). Doxycycline induction was performed in Flp-In To-REx 293 cells transfected with a pcDNA5 empty vector as a negative control. The cell pellets were lysed and centrifuged for 15 min at 20,000 *g* at 4°C, and the supernatant was transferred to a fresh tube. Equal amounts of protein were incubated with anti-VSV-G agarose beads (Sigma) at 4°C for 3 h on a rotating wheel. After incubation, samples were washed four times with immunoprecipitation buffer. Proteins were eluted from the beads by vortexing and boiling for 7 min in SDS buffer. Raf1 interacting proteins were detected and analyzed as previously described. Briefly, protein samples were digested with trypsin overnight at 37°C. Then, the peptide mixture was desalted and concentrated using a Peptide Microtrap (MW0.5–50 kDa, 0.5mm × 2 mm, Michrom Bioresources, CA, USA). The prepared sample (10 μL) was loaded onto a hybrid linear ion trap (LTQ) Orbitrap mass spectrometer (Thermo Fisher Scientific, Waltham, MA, USA) equipped with ADVANCE Spray Source (Michrom Bioresources, CA) and reversed-phase capillary column (0.1 mm × 150 mm, packed with 5 μm 100Å Magic C18 resin, Michrom Bioresources, CA, USA) with an auto-sampler (HTS-PAL, CTC Analytics, Zwingen, Switzerland) at a flow rate of 1 μL/min for 15 min. Other parameters and conditions were as previously described [30].

### Western blot analysis

We performed SDS-PAGE and immunoblotting as previously described [31]. For each sample, 30 μg of protein was separated by SDS-PAGE on 10–15% gels and transferred onto polyvinylidene difluoride membranes. Membranes were blocked in skimmed milk overnight at 4°C before incubating with primary antibodies followed by peroxidase-conjugated anti-mouse or anti-rabbit IgGs. The epitopes were detected using an enhanced chemiluminescence western blot detection kit.

### GO enrichment and KEGG pathway analysis

GO enrichment and KEGG pathway analysis were performed using the DAVID (https://david.ncifcrf.gov/) online tool. A P value of <0.05 was considered statistically significant. GO analysis revealed a significant enrichment in biological processes (BP), molecular function (MF), and cell component (CC) terms. KEGG (http://www.genome.jp/kegg/) systematically analyzed gene functions and linked genomic information with higher-order functional information. Additional functional annotations of the identified proteins were performed using the Ingenuity pathway analysis software.

### Integration of the protein–protein interaction (PPI) network

The Search Tool for the Retrieval of Interacting Genes (STRING) database is an online tool designed for evaluating protein–protein interaction (PPI) information. STRING (version 10.0) covers 9,643,763 proteins from 2031 organisms. To evaluate the interactive relationships of Raf1-interacting proteins, we mapped Raf1 to STRING. We only experimentally validated interactions with a combined score of >0.9, which were selected as significant. Then, PPI networks were constructed using Cytoscape software (version 3.4.0).

## SUPPLEMENTARY MATERIALS TABLES





















## Abbreviations

Raf: RAF proto-oncogene serine/threonine-protein kinase
MAPK: mitogen-activated protein kinase
MEK: mitogen-activated protein kinase kinase
ERK: extracellular signal regulated kinase
PP2A: protein phosphatase 2A
HSP90: heat shock protein 90
CDC37: cell division cycle 37
PAK: p21-activated kinase
STK3: serine/threonine kinase 3
PKC: protein kinase C
LC-MS: liquid chromatograph mass spectrometer
AKT: serine/threonine kinase 1
AIF: apoptosis inducing factor
FAR1: fatty acyl-CoA reductase1
KAP1: transcription intermediary factor 1-beta
RCN1/2: reticulocalbin-1/2
TIM50: mitochondrial import inner membrane translocase subunit TIM50
ANT2: ADP/ATP translocase 2
BAG4: BAG family molecular chaperone regulator 4
GRP78: 78 kDa glucose-regulated protein
IRS4: insulin receptor substrate 4
RUVB1: RuvB-like 1
RUVB2: RuvB-like 2
AKAP8L: A-kinase anchor protein 8-like
HAX1: HCLS1-associated protein X-1
CDK1: cyclin-dependent kinase 1
PARP: poly (ADP-ribose) polymerase 1
PSMC2: 26S protease regulatory subunit 7
SMC3: structural maintenance of chromosomes protein 3
NDUFA4: cytochrome c oxidase subunit NDUFA4.
