# Supplementary material for: Decoding the full picture of Raf1 function based on its interacting proteins
Source: Oncotarget. 2017 Jul 18;8(40):68329–37. doi: 10.18632/oncotarget.19353 (PMC5620260; doi:10.18632/oncotarget.19353)
Supplement: Supplementary file 1 [file oncotarget-08-68329-s001.pdf]

Decoding the full picture of Raf1 function based on its interacting proteins

SUPPLEMENTARY MATERIALS

Supplementary Table 1: Summary of 198 interactions of Raf1.

See Supplementary File 1

Supplementary Table 2: 69 interactions of Raf1 in STRING database.

See Supplementary File 2

Supplementary Table 3: Gene ontology analysis (molecular function) of Raf1 interacting proteins.

See Supplementary File 3

Supplementary Table 4: Gene ontology analysis (biological process) of Raf1 interacting proteins.

See Supplementary File 4

Supplementary Table 5: Gene ontology analysis (cellular compartment) of Raf1 interacting proteins.

See Supplementary File 5

Supplementary Table 6: KEGG pathway analysis of Raf1 interacting proteins.

See Supplementary File 6

Supplementary Table 7: Ingenuity Diseases and Bio Functions analysis of Raf1 binding proteins.

See Supplementary File 7

Supplementary Table 8: 14 binding proteins of Raf1 in Ingenuity Knowledge Base.

See Supplementary File 8

Supplementary Table 9: Ingenuity Canonical Pathways analysis of Raf1 interacting proteins.

See Supplementary File 9
